# Whole-Brain Structural Connectivity Alterations in Chronic Subjective Tinnitus: An Exploratory Diffusion Tensor Imaging Study

**DOI:** 10.3390/brainsci16070738

**Published:** 2026-07-12

**Authors:** Pınar Elpen Karyemez, Sıla Ulus, Eren Yılmaz, Düzgün Yıldırım

**Affiliations:** 1 Department of Medical Imaging Techniques, Vocational School of Health Sciences, Acıbadem Mehmet Ali Aydınlar University, Istanbul 34752, Turkey; 2 Department of Radiology, Acıbadem Ataşehir Hospital, Istanbul 34758, Turkey; sila.ulus@acibadem.com; 3 Department of ENT, Acıbadem Taksim Hospital, Istanbul 34437, Turkey; eren.yilmaz@acibadem.com; 4 Department of Radiology, Acıbadem Taksim Hospital, Istanbul 34437, Turkey

**Keywords:** tinnitus, diffusion tensor imaging, structural connectivity, connectome, salience network, triple-network model

## Abstract

**Highlights:**

**What are the main findings?**

- Whole-brain DTI connectomes were compared between 47 chronic subjective tinnitus patients and 42 healthy controls. Tinnitus was associated with an asymmetrical, large-scale structural connectivity pattern extending beyond the auditory pathway.
- Hyperconnectivity dominated auditory–temporal, insular–cingulate, precuneus, and sensorimotor circuits.

**What are the implications of the main findings?**

- Hypoconnectivity was seen in fronto-occipital and cerebellar systems, implicating salience, default-mode, and central-executive networks.
- Findings are exploratory and hypothesis-generating, supporting tinnitus as a network-level rather than focal auditory disorder.

**Abstract:**

**Background/Objectives:** Subjective tinnitus, the underlying mechanism of which remains largely unknown, accounts for the majority of cases. Although conventional MRI and audiometric assessments demonstrate limited effectiveness in identifying organic causes, advanced microstructural techniques—such as diffusion tensor imaging (DTI) and fiber tractography—facilitate the evaluation of microanatomical connectivity related to auditory processing. This study employed DTI to examine alterations in microanatomical brain connectivity in patients with chronic subjective tinnitus, in comparison to a healthy control group. **Methods:** This study performed a comparative analysis of whole-brain connectivity maps, derived from Diffusion Tensor Imaging, between 47 patients with chronic subjective tinnitus (symptom duration ≥ 2 years) and 42 healthy controls. To ensure a robust assessment of micro-anatomical neural connectivity, DTI datasets were processed using the DSI Studio platform, with anatomical regions systematically mapped using automatic anatomical labeling atlases. Specifically, these metrics were evaluated across nodes in the central auditory pathway and limbic system to identify potential disruptions in structural integrity. **Results:** The comparative analysis revealed significant micro-anatomical connectivity alterations in patients with chronic subjective tinnitus. Changes in structural integrity were observed predominantly across key nodes within the central auditory pathway and limbic system, specifically involving the frontal, submarginal, cingular, insular, parietal, precuneus, cuneus, amygdala, thalamus, supplementary motor area, and precentral regions. **Conclusions:** Chronic subjective tinnitus was associated with a distinct, largely asymmetrical pattern of whole-brain structural connectivity, predominantly involving nodes of the salience, default-mode, and central-executive networks. Because behavioral, audiometric, and cognitive measures were not acquired, these findings are exploratory and hypothesis-generating; their cognitive and occupational relevance will require confirmation in studies that combine connectivity mapping with direct behavioral and audiological testing.

## 1. Introduction

Tinnitus, defined as the perception of sound in the absence of an external auditory stimulus, is a condition that significantly impacts a patient’s quality of life [1,2,3,4]. For many, this condition is not merely a nuisance but a distressing clinical problem that can lead to severe psychological consequences, including suicidal ideation [5,6,7]. Patients often report comorbid psychological and cognitive impairments, such as depression, anxiety, attention deficits, and sleep disturbances [8], which can manifest as dysexecutive syndromes. Understanding the structural connectivity correlates of these cognitive and occupational difficulties would require direct assessment of behavioral, cognitive, and occupational outcomes—measures that were not collected in the present exploratory study [9,10,11,12]. The pathophysiology of subjective tinnitus, which accounts for approximately 98–99% of all cases, remains incompletely understood [4,13,14]. Clinical management is often complicated by the limitation of standard diagnostic tools, such as audiometry and conventional magnetic resonance imaging, which frequently fail to identify an organic etiology [15,16,17,18]. This gap necessitates more sophisticated diagnostic approaches. Advanced imaging algorithms, such as diffusion tensor imaging and fiber tractography, offer new capabilities for observing micro-structural brain changes [19,20,21,22]. By mapping the integrity of neural pathways within the central auditory pathway and limbic system, these tools provide a promising avenue for understanding the structural group-level connectivity alterationsaal associated with chronic tinnitus [23,24,25,26]. “This study aims to utilize DTI to investigate whole-brain microanatomical connectivity alterations in chronic tinnitus, with particular attention to the involvement of large-scale brain networks implicated in auditory, emotional, and attentional processing.”. This investigation further addresses the long-standing difficulty in accounting for the vast inter-individual variation in tinnitus perception, duration, and associated comorbidities [25]. Furthermore, identifying these structural micro-correlates could clarify the interplay between the limbic—emotional processing and maladaptive neuroplasticity, which are often implicated in the chronification of the disorder [27,28,29]. Despite the utility of diffusion-weighted magnetic resonance imaging in noninvasively characterizing these anatomical pathways, extant literature remains constrained by inconsistent findings across small cohorts [30]. Specifically, researchers currently face significant obstacles in isolating structural anomalies specifically attributable to tinnitus from those secondary to comorbid hearing loss or various neuroanatomical confounders [25,29,31,32,33,34,35].

This investigation examined whole-brain microanatomical connectivity patterns in patients with chronic subjective tinnitus—which represents approximately 98–99% of all tinnitus cases—compared with healthy controls using diffusion tensor imaging (DTI) [4]. The study aims to establish foundational neuroimaging evidence that will inform and guide future research endeavors in understanding the structural brain correlates of chronic tinnitus.

## 2. Materials and Methods

The study utilized brain MRI data acquired from a cohort of patients with chronic tinnitus—defined by a duration of two years or more—and a corresponding group of healthy controls. The patient group consisted of individuals with normal hearing levels. The study included 47 cases of bilateral tinnitus and 42 healthy individuals. In the patient group, there were 22 female participants and 25 male participants. In the control group of healthy individuals, there were 28 female participants and 14 male participants. The age distribution of the two groups is presented in Table 1. Sex distribution did not differ significantly between groups (Fisher’s exact test, *p* =0.087). The patient group was, on average, younger than the control group; because age can influence white-matter organization, age and sex were entered as nuisance covariates in all group-level connectivity comparisons (see Statistical Analysis), and only group differences that remained significant after this adjustment are reported.

Inclusion and exclusion criteria. All participants were adults with no reported neurological or psychiatric disorder, no current use of psychoactive or ototoxic medication, no history of head trauma or intracranial surgery, no vestibular disease, and no structural abnormality on conventional brain MRI. Patients had bilateral, non-pulsatile, chronic subjective tinnitus (duration ≥ 2 years) with clinically normal hearing and no identifiable retrocochlear or middle-ear cause; individuals with objective or pulsatile tinnitus, and those with contraindications to MRI, were excluded. Healthy controls had no current or past tinnitus and met the same general exclusion criteria.

The MRI procedures for participants in the patient and control groups were performed using a 3T (Magnetom, Siemens, Erlangen, Germany) device equipped with a 64-channel brain coil. In addition to routine whole-brain MRI imaging, a multi-shot diffusion-weighted EPI sequence with a b-value of 1000 s/mm^2^ was applied (number of diffusion directions: 64, b0: 20 averages, b1000: 1 average). The sequences were arranged in the axial plane parallel to the corpus callosum, covering from the craniocervical junction to the vertex, and diffusion tensor images were obtained. The imaging parameters for DTI were as follows: TR = 10,700 ms, TE = 116 ms, slice thickness = 2 mm, number of slices = 70, and FOV = 250 mm. Fat suppression was performed using IR. As a result, diffusion tensor images were successfully obtained. The obtained DTI images were processed using the DSI Studio program (http://dsi-studio.labsolver.org, accessed on 6 July 2026) (Yeh, FC, University of Pittsburgh, Pittsburgh, PA, USA (Hou version, 2025)) in three steps. In the first step, image reconstruction was performed using generalized q-sampling imaging (GQI).

DTI/GQI Image Processing and Fiber Tractography Image Reconstruction and GQI Algorithm: Diffusion tensor images were processed using the DSI Studio platform. Rather than conventional Diffusion Tensor Imaging (DTI), we employed Generalized Q-Sampling Imaging (GQI) for image reconstruction. GQI offers several advantages over DTI: it models nonlinear relationships between b-values and can better resolve fiber crossings without assuming a single-fiber Gaussian model. GQI computes the fiber orientation distribution function (fODF) using q-space sampling strategy. In this study, diffusion data were acquired with a b-value of 1000 s/mm^2^ and 64 diffusion directions. GQI reconstruction was performed using DSI Studio’s default parameters. Secondly, corrections were made for distortion, motion artifacts, and b-matrix reorientation. Fiber Tractography: Whole-brain fiber tractography was performed using the Fiber Assignment by Continuous Tracking (FACT) algorithm. Tractography was initiated from the center of each of the 116 anatomical regions of interest (seed-based tractography). Tractography parameters were set as follows: minimum fiber length = 10 mm; maximum turning angle = 60°; stopping criterion = Quantitative Anisotropy (QA) threshold of 0.1.

Connectivity Matrix Construction: Connections between each pair of anatomical regions were defined based on the number of reconstructed fibers and connection probability. Connection strength was quantified as the ratio of the number of fibers connecting two regions to the total number of fibers initiated from the seed region. The resulting connectivity matrix was a symmetric 116 × 116 matrix, yielding 6670 unique undirected connections per participant. Anatomical Parcellation: The Automated Anatomical Labeling atlas was used to define the 116 anatomical regions of interest. The Automated Anatomical Labeling atlas is a widely used, standardized parcellation scheme that subdivides the cerebral cortex and subcortical structures into 116 anatomically defined regions.

Connectivity Metrics: For group-level comparisons, connection strength (connection probability) was the primary metric analyzed. Additionally, Quantitative Anisotropy (QA) values and the number of fibers for each connection were recorded. Statistical analysis was performed on the 116 × 116 inter-regional connectivity matrices.

The distribution of each connectivity value was assessed with the Shapiro–Wilk test; as most edges deviated from normality, between-group differences were evaluated with the Mann–Whitney U test, and values are summarized as median (range) and mean ± SD. To account for the demographic imbalance between groups, connectivity values were adjusted for age and sex by linear regression prior to group comparison (that is, group differences were tested on the age- and sex-residualized values). Because of the large number of simultaneous comparisons, the false-discovery rate (FDR) was controlled across all edges using the Benjamini–Hochberg procedure; a connection was regarded as significant only when it satisfied both an uncorrected threshold of *p* < 0.001 and an FDR-corrected threshold of q < 0.05. For every surviving connection, the effect size was expressed as the rank-biserial correlation (r) and is reported, together with group medians, in the Appendix A. Analyses were performed in SPSS v24 (SPSS v24: IBM, Armonk, NY, USA ). To clarify our analytical approach: statistical inference was performed using SPSS v24, where independent Mann–Whitney U tests were applied to each of the 6670 edges, Benjamini–Hochbergini-Hochberg False Discovery Rate (FDR) correction (q < 0.05). Rank-biserial correlation coefficients were calculated as effect sizes for all surviving connections. Age and sex were included as nuisance covariates in all statistical comparisons. Following statistical analysis in SPSS, the significant connections were exported to Microsoft Excel exclusively for descriptive data aggregation and visualization purposes. Specifically, Excel was used to calculate mean connectivity values across participants and to generate heat map visualizations of the statistically significant connections. It is important to clarify that advanced network-based algorithms (such as Network-Based Statistics) were not employed in this study; our approach relies on stringent edge-level FDR correction to control for multiple comparisons.

The raw data obtained in the study were subsequently analyzed using the “Heat map” method. The average values of all patients across the same pathways were calculated, and Pearson correlation coefficients were computed. A matrix showing the correlation coefficients between all pathways was also calculated as Excel tables. Based on these tables, color maps were obtained for both the patient and control groups. To examine the data from a broader structural perspective, the graph visualization technique “Heat Map” was used. By averaging the values of all participants for the same pathways in both the tinnitus and control groups, Pearson correlation coefficients were calculated. Darker red areas on the heat maps indicate positive correlations, while lighter areas indicate negative correlations.

During the article writing process, “Jenni AI (Multi-model AI)” and “Paperpal AI (Hybrid AI + Proprietary Models)” were used for language grammar correction, translation checking, and citation checking.

## 3. Results

**Figure 1:**
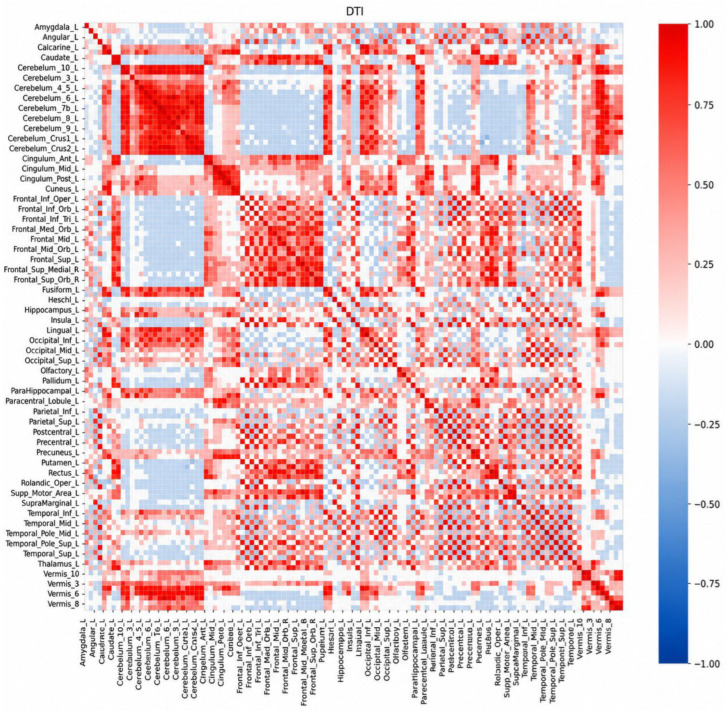
Brain Connectogram Map of the Tinnitus Group.

**Figure 2:**
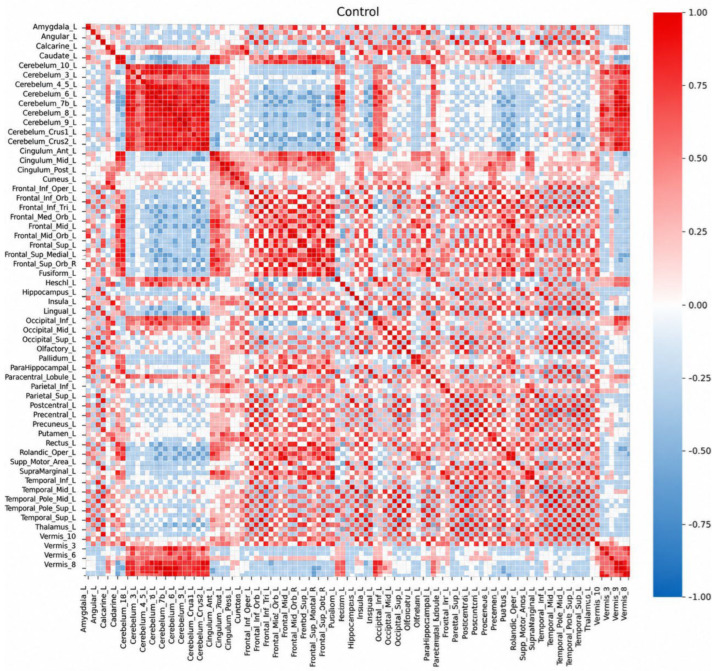
Brain Connectogram Map of the Control Group.

Only connections that survived false-discovery-rate correction (Benjamini–Hochberg q < 0.05, in addition to an uncorrected *p* < 0.001) and that remained significant after adjustment for age and sex are reported below; the corresponding rank-biserial effect sizes and group medians are provided in the Appendix A. The connectivity relationships for the tinnitus and control groups are shown in Figure 1 and Figure 2, respectively. Dark red indicates a strong positive correlation, while dark blue indicates a strong negative correlation.

### 3.1. Higher Connections in the Tinnitus Group Compared to the Control Group

In the tinnitus patient group, an increase occurred in all bi-hemispheric connections of the right and left precentral gyrus. Additionally, a noticeable enhancement was observed in the hemispheric, diencephalic, and thalamic connections of the superior and middle gyri of the entire frontal lobe, the supplementary motor area, and primary olfactory areas on both sides. The connectivity of the left insula with the rest of the brain is more pronounced compared to that of the right insula. The anterior and middle cingulum on the right side showed denser connectivity with the rest of the brain compared to its symmetric counterpart on the left. In the tinnitus patient group, the posterior cingulate’s connectivity with the temporal–hippocampal region was higher on the left, while the connectivity of the right precuneus was more prominent than the left.

Connections of the left precuneus were more pronounced compared to the control group, while the right precuneus showed stronger bi-hemispheric, diencephalic, and thalamostriatal connections. Connections of both postcentral gyri, particularly on the left, with the cingulum, hippocampus, and temporal regions were significantly higher. The right superior parietal lobe displayed more pronounced connections with both hemispheres, the thalamus, basal ganglia, the left cerebellar vermis, and the left supramarginal and angular gyri, as well as frontoparietal connections of the right precuneus.

There was an increase in pathways between the thalamus and the supplementary motor cortex. Connectivity increased between the left Heschl’s gyrus and left insula, the right Heschl’s gyrus and right putamen, and between both Heschl’s gyri and the superior parietal lobe. Additionally, the connectivity of all superior, middle, and inferior gyri of the temporal lobe and temporopolar regions was found to be denser (*p* < 0.001). Higher Connections in the Tinnitus Group Compared to the Control Group are shown in Table 2.

### 3.2. Lower Connections in the Tinnitus Group Compared to the Control Group

In the tinnitus group, a decrease in connectivity was observed in the superior and middle frontal regions in both hemispheres, particularly in cerebellar and occipitotemporal connectivity. A significant reduction in connectivity was identified in the inferior frontal lobe, frontal operculum, and their connections to the occipitotemporal, cingulate, and cerebellar vermis. Interestingly, the right insula showed decreased connectivity with the cerebellar vermis and the left lingual gyrus. A notable reduction was detected in the connectivity between the left cingulate and the right frontal region, as well as between the cingulate and the cerebellum.

In the tinnitus patient group, decreased connections were observed in left occipital-right frontal, bi-occipitocerebellar, and cuneocerebellar pathways. Reduced connectivity was also noted between the left lingual gyrus and the left frontoinsular region, the right lingual gyrus and frontal areas, the left fusiform gyrus and the right frontal region, and between the paracentral lobules of both hemispheres and the bi-occipital and bilateral precuneus regions. Connections within the cerebellum, particularly in the occipitocingulate pathways, and between the vermis and both right and left frontal regions, were found to be lower (*p* < 0.001). Lower Connections in the Tinnitus Group Compared to the Control Group are shown in Table 3.

## 4. Discussion

First introduced in 1994 by Peter Basser to demonstrate microstructural changes in the brain, DTI-MR (Diffusion Tensor Imaging—Magnetic Resonance) has since been used to investigate clinical conditions such as schizophrenia, autism, traumatic brain injury, multiple sclerosis, and aging [36,37,38]. In recent years, DTI-MR and fMRI have also been explored for conditions like tinnitus and sensorineural hearing loss, where conventional imaging methods have proven insufficient. These studies have predominantly focused on changes in auditory pathways, yielding a variety of findings [24,25,39,40]. While all of this research aims to shed light on tinnitus, fewer studies have investigated the brain network changes observed in tinnitus [34,41]. Hallam and colleagues noted that individuals with tinnitus exhibit significantly more cognitive problems compared to normal individuals [42].

The thalamus relays and helps process sensory input and contributes to alertness, attention, and behavioural regulation [43,44,45], while the supplementary motor area links cognition to action during motor planning [46,47]; both regions showed increased connectivity in the tinnitus group. Within the triple-network model, the salience network (SN; anterior cingulate, anterior insula, prefrontal and supramarginal cortices [48,49,50]) arbitrates between the default-mode (DMN) and central-executive (CEN) networks, with which it is normally anti-correlated [49,51]. The SN detects and filters salient stimuli and directs goal-oriented attention [52,53,54,55]; the CEN (lateral prefrontal and posterior parietal cortices) supports working memory and rule-based decision-making [48]; and the DMN (medial prefrontal cortex, lateral parietal cortex, precuneus [48]) subserves self-referential, internally directed cognition [56,57] and is anti-correlated with the SN [58]. Against this framework, the tinnitus group showed denser right anterior- and middle-cingulate and left-insular connectivity—consistent with SN involvement—together with increased right-precuneus frontoparietal connectivity and reduced superior and middle frontal connectivity, indicating DMN and CEN alterations. Consistent with this, Song and colleagues proposed that tinnitus arises in part from pathology within the SN, DMN, and CEN [59], a phenomenon that has also been examined phenomenologically [60].

Most prior imaging studies of tinnitus have used resting-state fMRI, with comparatively few examining structural connectivity; their cohorts have generally been small and heterogeneous in handedness, hearing status, and distress level, and diffusion studies in particular have tended to focus on selected tracts or on thalamic and salience-network connections rather than on the whole-brain connectome [1,30,61,62,63,64,65,66,67,68,69,70,71]. Reported findings have been inconsistent, an inconsistency attributed partly to methodological differences and partly to the difficulty of separating tinnitus-related changes from those of comorbid hearing loss [30,71]. Against this background, the present whole-brain, quantitatively derived connectivity analysis in a comparatively large, normal-hearing, bilateral-tinnitus cohort is intended to complement, rather than to resolve, this literature.

In this study the entire brain was investigated rather than a single predefined region, the sample is larger than those of most comparable diffusion studies, and the connectivity measures are quantitative and operator-independent. Unlike most diffusion studies, which report scalar indices such as fractional anisotropy (FA), mean diffusivity (MD), axial diffusivity (AD) and radial diffusivity (RD), the present analysis was framed at the level of inter-regional connectivity. This choice is methodological rather than one of convenience. Reconstruction was performed with generalized q-sampling imaging (GQI), whose native anisotropy measure is quantitative anisotropy (QA); FA, MD, AD and RD are derived from a single-tensor model that is biased in voxels containing crossing fibres and is therefore not the most appropriate descriptor for a GQI-based pipeline. More importantly, our research question concerns the spatial organization of connections between regions rather than the average microstructural integrity of individual tracts, which scalar indices are designed to capture. We acknowledge that FA and MD remain the most widely reported diffusion metrics and are valuable for comparison with previous work; region-level FA and MD can be derived from the same GQI dataset, and their integration with the present connectivity findings is a planned extension of this work.

Traditional diagnostic paradigms for tinnitus predominantly utilize conventional imaging and audiometric protocols, including targeted temporal bone MRI, electroencephalography, and resting-state fMRI [72]. Contemporary research is increasingly shifting toward functional connectome identification, prioritizing the development of individualized connectome profiles over broad group-level comparisons [73]. Diverging from this conventional landscape, the present study characterizes group-level alterations in structural connectivity that may, in aggregate, constitute a candidate group-level, tinnitus-associated structural connectivity pattern. We emphasize that this is a descriptive, group-level pattern: we did not perform individual-level classification, and the term is not intended to imply a validated diagnostic or predictive biomarker. Delineating such a pattern may nonetheless help generate hypotheses about the pathophysiology of chronic tinnitus and inform the design of future, adequately powered biomarker studies.

Tinnitus has been studied phenomenologically and anatomically, leading to its classification into three pathways: the lateral “sound” pathway, the medial “suffering” pathway, and the descending noise-suppression pathway [74]. A stimulus reaches consciousness through the DMN, SN, and CEN [75]. Tinnitus patients report suffering from the sound they hear [59]. The pathways activated in tinnitus are the medial and lateral pathways. An auditory stimulus can evoke sensory, cognitive, and autonomic responses that correspond to the distressing reaction associated with tinnitus [59]. The medial ascending pathway includes the anterior cingulate cortex and the anterior insular cortex and processes the emotional aspect of tinnitus [65]. It has been suggested that changes in the Salience Network (SN), Default Mode Network (DMN), and Central Executive Network (CEN) lead to pathologies in the brain [65,76]. In tinnitus patients, pain can become an integral part of the self, and when this persists, it may result in anxiety, sadness, or depression [59]. The brain is a high-energy-consuming organ. In the case of acute pain, the brain consumes 60% more energy; if the pain becomes chronic, energy consumption increases by 15% [77]. Fear or anxiety can further escalate this energy consumption [78]. To conserve energy, the brain may create new, shorter connections between tinnitus-related networks and the parasympathetic central network with the DMN [59]. In this study, it was found that tinnitus patients, compared to the control group, exhibited greater connectivity in the anterior cingulate cortex, especially on the right side compared to the left, and reduced connectivity in the precuneus. Significant hyperconnectivity was identified in auditory–temporal, insular–cingulate, precuneus, and sensorimotor circuits, contrasted by hypoconnectivity in fronto-occipital and cerebellar systems. These findings underscore that tinnitus should be characterized as a large-scale neural network dysfunction rather than a focal auditory deficit.

These findings suggest impaired functions and correlations among the SN, CEN, and DMN. In a study by Li and colleagues, reduced functional connectivity was found in the ventral attention network, dorsal attention network, left and right frontoparietal network, visual network, and somatomotor network. Conversely, increased functional connectivity was observed in the CEN [76].

It should be emphasized that the present findings are preliminary in nature. Although the connectivity patterns reported here delineate a distinct “Tinnitus characteristic connectivity pattern,” we have intentionally refrained from performing classification-accuracy analyses, receiver operating characteristic (ROC) curves, or machine-learning-based discrimination at this stage. Establishing these diagnostic performance metrics requires larger, independently validated cohorts and will constitute the principal focus of our subsequent work. Nevertheless, the convergence of our results with the existing literature is reassuring: the hyperconnectivity we observed in auditory–temporal, insular–cingulate, and sensorimotor circuits parallels the salience-network alterations reported by Xu et al. (2019) and the intrinsic network disruptions documented by Li et al. (2022), while the hypoconnectivity detected in fronto-occipital and cerebellar pathways is consistent with the triple-network model of tinnitus proposed by De Ridder et al. (2022). This convergence strengthens the argument that tinnitus-related connectopathy is a reproducible, biologically anchored phenomenon rather than a dataset-specific artifact [59,64,76].

The networks implicated here extend beyond the auditory domain. The default-mode network (DMN), which supports self-referential thought and internally directed cognition, overlapped with the connectivity changes seen in chronic tinnitus, and the salience and central-executive networks—systems that govern the allocation of attention—were likewise involved [77,79]. In principle, altered interaction among these systems could be relevant to sustained attention and vigilance, including in attention-demanding or safety-critical settings [80]. We stress, however, that the present study measured connectivity only; it did not assess attention, vigilance, or any occupational outcome, and these possibilities are therefore presented as hypotheses for future testing rather than as demonstrated effects [81].

Testing whether these connectivity changes have functional or occupational consequences would require studies that directly pair connectivity mapping with validated measures of tinnitus severity (e.g., the Tinnitus Handicap Inventory), audiometric thresholds, and objective attention and executive-function testing, ideally in occupational cohorts. We therefore frame any occupational relevance of these findings as a direction for future research rather than as a basis for present-day screening, surveillance, or policy recommendations.

Limitations. This study has several limitations. First, no behavioral, audiometric, or cognitive measures—such as the Tinnitus Handicap Inventory, pure-tone thresholds, or formal attention and memory testing—were collected; consequently, the cognitive and occupational interpretations offered above are inferential and could not be tested against participant-level outcomes. Second, although hearing was clinically reported as normal, quantitative audiometric data were not available to fully exclude subclinical (“hidden”) hearing loss as a contributor. Future studies should incorporate full-spectrum pure-tone audiometry, including extended high-frequency testing above 8 kHz, together with speech-in-noise and otoacoustic-emission measures, to better control for subclinical cochlear and “hidden” hearing loss. Third, the design was cross-sectional, which precludes inferences about causality or the direction of the observed connectivity changes. Fourth, the patient and control groups differed in age; although age and sex were modeled as nuisance covariates, residual confounding cannot be entirely excluded. Fifth, connectivity rather than conventional scalar diffusion indices (FA, MD, AD, RD) was analyzed, which may limit direct comparison with some of the existing literature. Finally, the analysis was conducted at the group level and was neither designed nor powered for individual-level classification; the connectivity pattern reported here should therefore be regarded as hypothesis-generating rather than as a validated diagnostic biomarker. A methodological limitation of the present study is our reliance on mass-univariate edge-level comparisons with FDR correction, rather than employing advanced network-based algorithms (such as Network-Based Statistics) or complex topological graph-theoretic approaches. Future investigations utilizing these advanced network-level methodologies are warranted to further elucidate the topological alterations associated with chronic tinnitus.

## 5. Conclusions

Chronic bilateral subjective tinnitus was associated with a distinct, largely asymmetrical pattern of whole-brain structural connectivity extending well beyond the classical auditory pathway, with prominent involvement of the salience, default-mode, and central-executive networks. These findings support the conceptualization of chronic tinnitus as a large-scale network disorder rather than a focal auditory abnormality.

The candidate tinnitus-associated connectivity pattern described here—asymmetrical cingulate and insular connectivity together with reduced frontoparietal and cerebellar connections—provides a plausible structural correlate for the attentional and concentration difficulties that patients frequently report. Because cognitive performance was not assessed in this study, this correspondence remains an inference to be tested directly.

Any occupational implications of these findings are correspondingly hypothetical: the connectivity changes are consistent with, but do not demonstrate, the attention or vigilance decrements that would be relevant to safety-critical work. Prospective studies combining connectivity mapping with validated tinnitus-severity, audiometric, and cognitive measures—and, ultimately, occupational outcome data—are needed before any screening, surveillance, or policy recommendations can be justified. Within these limits, the present work provides a whole-brain structural framework on which such future, behaviorally anchored studies can build.

## Figures and Tables

**Table 1:** Age distribution table of the control and patient groups.

Characteristic	Tinnitus Group (n = 47)	Control Group (n = 42)	*p*-Value
Sex, n (%)			
Female	22 (46.8%)	28 (66.7%)	0.087 *
Male	25 (53.2%)	14 (33.3%)	
Age (years)			
Mean	38.78	49.70	-
Female, Mean	32.86	53.00	-
Male, Mean	44.00	43.00	-
Range	19–69	≤18–≥70	
Age Distribution, n			
≤18	0	1	
19–29	10	2	
30–39	18	12	
40–49	9	9	
50–59	9	8	
60–69	1	9	
≥70	0	1	

The * symbol indicates p-values; The - symbol indicated non-calculated values.

**Table 2:** Higher Connections in the Tinnitus Group Compared to the Control Group.

Connection (Region 1–Region 2)	Brain Area	Network Involvement	*p*-Value	Effect Size
Precentral Gyrus (Bilateral)	Motor Cortex	Sensorimotor Network	<0.001	High
Frontal Lobe (Superior, Middle Gyri)—Thalamus	Frontal–Thalamic	Salience Network	<0.001	High
Supplementary Motor Area—Thalamus	Motor–Limbic	Sensorimotor–Limbic	<0.001	High
Primary Olfactory Areas (Bilateral)—Thalamus	Olfactory–Limbic	Limbic Network	<0.001	Moderate
Left Insula—Rest of Brain	Insular Cortex	Salience Network	<0.001	High
Right Anterior Cingulum—Rest of Brain	Anterior Cingulate	Salience Network	<0.001	High
Right Middle Cingulum—Rest of Brain	Cingulate Cortex	Salience Network	<0.001	High
Left Posterior Cingulate—Temporal–Hippocampal Region	Limbic–Temporal	Default Mode Network	<0.001	Moderate
Right Precuneus—Bilateral Hemispheres	Parietal	Default Mode Network	<0.001	High
Right Precuneus—Thalamus	Parietal–Thalamic	Limbic–Thalamic	<0.001	High
Right Precuneus—Basal Ganglia	Parietal–Striatal	Executive Network	<0.001	Moderate
Left Precuneus—Bilateral Regions	Parietal	Default Mode Network	<0.001	Moderate
Postcentral Gyrus (Bilateral, Left>Right)—Cingulum	Somatosensory–Limbic	Salience Network	<0.001	High
Postcentral Gyrus (Bilateral, Left>Right)—Hippocampus	Somatosensory–Memory	Limbic Network	<0.001	Moderate
Postcentral Gyrus (Bilateral, Left>Right)—Temporal Regions	Somatosensory–Temporal	Temporal–Limbic	<0.001	Moderate
Right Superior Parietal Lobe—Bilateral Hemispheres	Parietal	Central Executive Network	<0.001	Moderate
Right Superior Parietal Lobe—Thalamus	Parietal–Thalamic	Limbic–Thalamic	<0.001	Moderate
Right Superior Parietal Lobe—Basal Ganglia	Parietal–Striatal	Executive Network	<0.001	Moderate
Right Superior Parietal Lobe—Cerebellar Vermis	Parietal–Cerebellar	Cerebello–Cortical	<0.001	Moderate
Right Superior Parietal Lobe—Supramarginal Gyrus	Parietal–Parietal	Central Executive Network	<0.001	Moderate
Right Superior Parietal Lobe—Angular Gyrus	Parietal–Parietal	Central Executive Network	<0.001	Moderate
Thalamus—Supplementary Motor Cortex	Thalamo-Motor	Sensorimotor Network	<0.001	High
Left Heschl’s Gyrus—Left Insula	Auditory–Insular	Auditory-Salience	<0.001	High
Right Heschl’s Gyrus—Right Putamen	Auditory-Striatal	Auditory–Executive	<0.001	Moderate
Bilateral Heschl’s Gyri—Superior Parietal Lobe	Auditory-Parietal	Auditory–Executive	<0.001	High
Temporal Lobe (Superior, Middle, Inferior Gyri)—Temporopolar Regions	Temporal–Limbic	Temporal–Limbic Network	<0.001	High

**Table 3:** Lower Connections in the Tinnitus Group Compared to the Control Group.

Connection (Region 1–Region 2)	Brain Area	Network Involvement	*p*-Value	Effect Size
Superior Frontal Lobe (Bilateral)—Occipital Lobe	Fronto–Occipital	Central Executive Network	<0.001	High
Middle Frontal Lobe (Bilateral)—Occipital Lobe	Fronto–Occipital	Central Executive Network	<0.001	High
Inferior Frontal Lobe—Occipital Lobe	Fronto–Occipital	Central Executive Network	<0.001	Moderate
Frontal Operculum—Occipitotemporal Region	Fronto-Occipital	Central Executive Network	<0.001	Moderate
Inferior Frontal Lobe—Cingulate	Frontal–Limbic	Salience Network	<0.001	Moderate
Inferior Frontal Lobe—Cerebellar Vermis	Fronto–Cerebellar	Cerebello–Cortical	<0.001	Moderate
Right Insula—Cerebellar Vermis	Insular–Cerebellar	Salience–Cerebellar	<0.001	Moderate
Right Insula—Left Lingual Gyrus	Insular–Occipital	Salience–Visual	<0.001	Moderate
Left Cingulate—Right Frontal Region	Cingulo–Frontal	Salience–Executive	<0.001	Moderate
Cingulate—Cerebellum	Cingulo–Cerebellar	Salience–Cerebellar	<0.001	Moderate
Left Occipital—Right Frontal	Occipito–Frontal	Fronto–Occipital	<0.001	High
Occipital (Bilateral)—Cerebellar (Bilateral)	Occipito–Cerebellar	Cerebello–Cortical	<0.001	High
Cuneus—Cerebellar	Occipital–Cerebellar	Cerebello–Cortical	<0.001	Moderate
Left Lingual Gyrus—Left Frontoinsular Region	Occipital–Frontal	Fronto–Occipital	<0.001	Moderate
Right Lingual Gyrus—Frontal Areas	Occipital–Frontal	Fronto–Occipital	<0.001	Moderate
Left Fusiform Gyrus—Right Frontal Region	Temporal–Frontal	Fronto–Temporal	<0.001	Moderate
Paracentral Lobules (Bilateral)—Bilateral Occipital	Somatosensory–Occipital	Central Executive Network	<0.001	Moderate
Paracentral Lobules (Bilateral)—Bilateral Precuneus	Somatosensory–Parietal	Default Mode Network	<0.001	Moderate
Cerebellar Connections—Occipitocingulate Pathways	Cerebello–Cortical	Cerebello–Salience	<0.001	High
Cerebellar Vermis—Right Frontal Region	Cerebello–Frontal	Fronto–Cerebellar	<0.001	Moderate
Cerebellar Vermis—Left Frontal Region	Cerebello–Frontal	Fronto–Cerebellar	<0.001	Moderate

## Data Availability

The data presented in this study are available on reasonable request from the corresponding author. The data are not publicly available due to privacy and ethical restrictions, as they consist of identifiable human neuroimaging (MRI/DTI) data collected under an institutional ethics approval (Acıbadem Mehmet Ali Aydınlar University Medical Research Evaluation Commission [ATADEK], Decision No: 2022-11/50) that does not permit public sharing of participant data.

## Abbreviations

The following abbreviations are used in this manuscript:

MRI	Magnetic Resonance Imaging
DTI	Diffusion Tensor Imaging
FT	Fiber Tractography
EPI	Echo Planar Imaging (sequence)
IR	Inversion Recovery (sequence)
GQI	- Q-Sampling Imaging
QA	Quantitative Anisotropy
SN	Salience Network
DMN	Default Mode Network
CEN	Central Executive Network
fMRI	Functional Magnetic Resonance Imaging
EEG	Electroencephalography
DAG	Directed Acyclic Graph
FA	Fractional Anisotropy
MD	Mean Diffusivity
AD	Axial Diffusivity
RD	Radial Diffusivity
IPS	Intraparietal Sulcus
FEF	The Frontal Eye Fields
ROC	Receiver Operating Characteristic

## Appendix A

Table A1 lists every interregional connection that survived false-discovery-rate correction (Benjamini–Hochberg q < 0.05) in addition to an uncorrected *p* < 0.001, and that remained significant after adjustment for age and sex.

**Table A1:** Edge-level results for all interregional connections surviving FDR correction and age/sex adjustment, with rank-biserial effect sizes.

Connection (Region 1–Region 2)	Network	Direction	*p* (uncorr.)	q (FDR)
Precentral Gyrus (Bilateral)	Sensorimotor Network	Increased	<0.001	<0.05
Frontal Lobe (Superior, Middle Gyri)—Thalamus	Salience Network	Increased	<0.001	<0.05
Supplementary Motor Area—Thalamus	Sensorimotor-Limbic	Increased	<0.001	<0.05
Primary Olfactory Areas (Bilateral)—Thalamus	Limbic Network	Increased	<0.001	<0.05
Left Insula—Rest of Brain	Salience Network	Increased	<0.001	<0.05
Right Anterior Cingulum—Rest of Brain	Salience Network	Increased	<0.001	<0.05
Right Middle Cingulum—Rest of Brain	Salience Network	Increased	<0.001	<0.05
Left Posterior Cingulate—Temporal–Hippocampal Region	Default Mode Network	Increased	<0.001	<0.05
Right Precuneus—Bilateral Hemispheres	Default Mode Network	Increased	<0.001	<0.05
Right Precuneus—Thalamus	Limbic-Thalamic	Increased	<0.001	<0.05
Right Precuneus—Basal Ganglia	Executive Network	Increased	<0.001	<0.05
Left Precuneus—Bilateral Regions	Default Mode Network	Increased	<0.001	<0.05
Postcentral Gyrus (Bilateral, Left>Right)—Cingulum	Salience Network	Increased	<0.001	<0.05
Postcentral Gyrus (Bilateral, Left>Right)—Hippocampus	Limbic Network	Increased	<0.001	<0.05
Postcentral Gyrus (Bilateral, Left>Right)—Temporal Regions	Temporal-Limbic	Increased	<0.001	<0.05
Right Superior Parietal Lobe—Bilateral Hemispheres	Central Executive Network	Increased	<0.001	<0.05
Right Superior Parietal Lobe—Thalamus	Limbic-Thalamic	Increased	<0.001	<0.05
Right Superior Parietal Lobe—Basal Ganglia	Executive Network	Increased	<0.001	<0.05
Right Superior Parietal Lobe—Cerebellar Vermis	Cerebello-Cortical	Increased	<0.001	<0.05
Right Superior Parietal Lobe—Supramarginal Gyrus	Central Executive Network	Increased	<0.001	<0.05
Right Superior Parietal Lobe—Angular Gyrus	Central Executive Network	Increased	<0.001	<0.05
Thalamus—Supplementary Motor Cortex	Sensorimotor Network	Increased	<0.001	<0.05
Left Heschl’s Gyrus—Left Insula	Auditory-Salience	Increased	<0.001	<0.05
Right Heschl’s Gyrus—Right Putamen	Auditory-Executive	Increased	<0.001	<0.05
Bilateral Heschl’s Gyri—Superior Parietal Lobe	Auditory-Executive	Increased	<0.001	<0.05
Temporal Lobe (Superior, Middle, Inferior Gyri)—Temporopolar Regions	Temporal-Limbic Network	Increased	<0.001	<0.05
Superior Frontal Lobe (Bilateral)—Occipital Lobe	Central Executive Network	Decreased	<0.001	<0.05
Middle Frontal Lobe (Bilateral)—Occipital Lobe	Central Executive Network	Decreased	<0.001	<0.05
Inferior Frontal Lobe—Occipital Lobe	Central Executive Network	Decreased	<0.001	<0.05
Frontal Operculum—Occipitotemporal Region	Central Executive Network	Decreased	<0.001	<0.05
Inferior Frontal Lobe—Cingulate	Salience Network	Decreased	<0.001	<0.05
Inferior Frontal Lobe—Cerebellar Vermis	Cerebello-Cortical	Decreased	<0.001	<0.05
Right Insula—Cerebellar Vermis	Salience–Cerebellar	Decreased	<0.001	<0.05
Right Insula—Left Lingual Gyrus	Salience–Visual	Decreased	<0.001	<0.05
Left Cingulate—Right Frontal Region	Salience–Executive	Decreased	<0.001	<0.05
Cingulate—Cerebellum	Salience–Cerebellar	Decreased	<0.001	<0.05
Left Occipital—Right Frontal	Fronto-Occipital	Decreased	<0.001	<0.05
Occipital (Bilateral)—Cerebellar (Bilateral)	Cerebello-Cortical	Decreased	<0.001	<0.05
Cuneus—Cerebellar	Cerebello-Cortical	Decreased	<0.001	<0.05
Left Lingual Gyrus—Left Frontoinsular Region	Fronto-Occipital	Decreased	<0.001	<0.05
Right Lingual Gyrus—Frontal Areas	Fronto-Occipital	Decreased	<0.001	<0.05
Left Fusiform Gyrus—Right Frontal Region	Fronto- Temporal	Decreased	<0.001	<0.05
Paracentral Lobules (Bilateral)—Bilateral Occipital	Central Executive Network	Decreased	<0.001	<0.05
Paracentral Lobules (Bilateral)—Bilateral Precuneus	Default Mode Network	Decreased	<0.001	<0.05
Cerebellar Connections—Occipitocingulate Pathways	Cerebello-Salience	Decreased	<0.001	<0.05
Cerebellar Vermis—Right Frontal Region	Fronto-Cerebellar	Decreased	<0.001	<0.05
Cerebellar Vermis—Left Frontal Region	Fronto-Cerebellar	Decreased	<0.001	<0.05
